# MicroRNA expression is associated with auditory dysfunction in workers exposed to ototoxic solvents and noise

**DOI:** 10.3389/fpubh.2022.958181

**Published:** 2022-09-20

**Authors:** Renata Sisto, Arturo Moleti, Pasquale Capone, Filippo Sanjust, Luigi Cerini, Giovanna Tranfo, Giulia Massini, Sara Buscema, Paolo Massimo Buscema, Pieranna Chiarella

**Affiliations:** ^1^Department of Occupational and Environmental Medicine, Epidemiology and Hygiene, Italian Workers Compensation Authority (INAIL), Rome, Italy; ^2^Department of Physics and NAST Centre, University of Roma Tor Vergata, Rome, Italy; ^3^Semeion, Research Center of Sciences of Communication, Rome, Italy

**Keywords:** painting, microRNA, auditory function, hearing level, otoacoustic emissions, artificial neural networks, auto contractive map

## Abstract

This study is part of a project on early hearing dysfunction induced by combined exposure to volatile organic compounds (VOCs) and noise in occupational settings. In a previous study, 56 microRNAs were found differentially expressed in exposed workers compared to controls. Here, we analyze the statistical association of microRNA expression with audiometric hearing level (HL) and distortion product otoacoustic emission (DPOAE) level in that subset of differentially expressed microRNAs. The highest negative correlations were found; for HL, with miR-195-5p and miR-122-5p, and, for DPOAEs, with miR-92b-5p and miR-206. The homozygous (*mut*) and heterozygous (*het*) variants of the gene hOGG1 were found disadvantaged with respect to the wild-type (*wt*), as regards the risk of hearing impairment due to exposure to VOCs. An unsupervised artificial neural network (auto contractive map) was also used to detect and show, using graph analysis, the hidden connections between the explored variables. These findings may contribute to the formulation of mechanistic hypotheses about hearing damage due to co-exposure to noise and ototoxic solvents.

## Introduction

The combined exposure to noise and ototoxic/neurotoxic substances in occupational settings represents a serious risk factor for the auditory function of workers. Noise-induced hearing loss is a well-known risk factor in occupational health, mainly related to hair cell damage in the basal region of the cochlea (1, 2). VOCs (i.e., toluene, styrene, xylene, and benzene) are neurotoxic chemical solvents used in specific working sectors, such as transportation, mining, construction, and manufacturing industries, which may also be present in glues, paints, varnishes, and cleaning products. It has been shown that persistent exposure to neurotoxic agents impairs the auditory function of professional workers, mainly causing damage to the inner ear. Several published papers demonstrated an increased hearing loss in workers simultaneously exposed to VOCs and noise compared to those exposed to noise only, confirming that the combination of the two agents seriously affects hearing (3–7).

The standard outcome variable for the evaluation of hearing dysfunction is the hearing level (HL) shift measured at a set of different frequencies by pure-tone audiometry. This outcome variable is clinically relevant as it integrates the adverse effects on the auditory system from the periphery to the auditory cortex. The HL is sensitive to the functionality of the cochlear receptor and, in particular, of the cochlear amplifier, identified with the outer hair cells (OHC) system, but it is also sensitive to the health status of the synapses and the whole retrocochlear auditory pathway. To distinguish the peripheral damage from the central one, distortion product otoacoustic emissions (DPOAEs) were also collected and analyzed. DPOAEs are acoustic signals generated in the cochlea as a nonlinear distortion response to acoustic stimuli, and measurable in the ear canal. Their level is strictly related to the effectiveness of the active feedback mechanism provided by the cochlear OHCs. Due to the cochlear nonlinearity, DPOAEs are evoked by two tones, f_1_ and f_2_, at several intermodulation distortion frequencies, and among them, the clinically most important is f_DP_ = 2f_1_-f_2_. The 2f_1_-f_2_ distortion product is nonlinearly generated within a cochlear region (whose spatial width is a function of the stimulus levels) near the cochlear place, where the f_2_ frequency is resonant; therefore, the corresponding DPOAE provides an objective test of the cochlear functionality at frequency f_2_.

To our knowledge, there are no previous studies regarding the association between microRNA expression and auditory variables in subjects professionally exposed to solvents. The data shown in this paper are part of a larger study aimed at assessing the early hearing dysfunction induced by the combined exposure to VOCs and noise in an occupational setting. In previous studies (3, 8), the association between exposure to VOCs and hearing dysfunction in painters was demonstrated, also showing that the outcome variables of audiological tests are significantly associated with oxidative stress. The ototoxicity of VOCs has also been well established by studies on animals, which provided crucial clues to understand the underlying molecular mechanisms, as reported in several papers (9–11). As the receptors are particularly sensitive to the damage induced by xenobiotics, ototoxicity represents the main target to analyze the neurotoxic effects induced by VOCs. The aim of this work is to evaluate the association between the hearing dysfunction assessed by audiometry and DPOAEs and the microRNA profiles previously identified in a cohort of workers exposed to VOCs.

MicroRNAs are short non-coding RNAs playing a role in the epigenetic regulation of gene expression, acting at a post-transcriptional level to modulate the protein-encoding genes (12, 13). MicroRNAs were first identified as novel biomarkers in the diagnosis and prognosis of serious diseases, including cancer, neurological disorders, and cardiovascular diseases (12–14). However, microRNAs have also been recognized as biomarkers of dose and early adverse effects in toxicological studies, showing a functional role in the environmental and occupational risk assessment (15). Our research group has recently demonstrated that the microRNA expression pattern of exposed workers is significantly affected by exposure to VOCs in the industrial painting setting (16). Using the same data set of exposed workers of the present study, a set of microRNAs was found differentially expressed in workers exposed to VOCs, with respect to a control group of non-exposed subjects (17), and the statistical association between the VOC exposure and the hearing dysfunction was demonstrated in the exposed subject subset (3). As the damaging effect due to VOC exposure, in general, to each xenobiotic, also depends on the individual capability of detoxification and resistance to oxidative stress, the polymorphism of two genes involved in the DNA repair (human 8-oxoguanine DNA N-glycosylase 1 (hOGG1) and X-Ray Repair Cross Complementing 1 (XRCC1) was also considered as a possible susceptibility index (18, 19).

## Methods

The subjects enrolled in this study are 17 male painters (11 roller-painters and 6 spray-painters, the latter being characterized by higher levels of exposure to both noise and VOCs), aged from 21 to 54 years (average 39 years). Serum samples were collected in the framework of the routine tests requested by sanitary surveillance protocols. The analysis of biological samples was described in detail elsewhere (17). Workers agreed to be enrolled in the study, giving their informed consent, according to the principles outlined in the Declaration of Helsinki. All procedures performed in this study involving human participants were in accordance with the ethical standards of our Institutional Committee and with the local ethical committee (Health Local Agency, ASL, Regione Marche).

The professional exposure to noise was assessed using wearable phonometers Quest DLX-1. The personal A-weighted daily noise exposure was evaluated, over the whole duration (8 h) of a typical working day.

All the VOC metabolites, mandelic acid (MA) and phenylglyoxylic acid (PGA), both metabolites of the ethylbenzene, methylhippuric acid (MHIPP, xylenes metabolite), SPMA, S-phenylmercapturic acid (SPMA, benzene metabolite), and S-benzyl mercapturic acid (SBMA, toluene metabolite) were measured in the end shift urine matrix. Cotinine was also determined as a metabolite of nicotine.

All values were divided by the concentration of urinary creatinine to normalize results for the dilution grade of urine (3, 19).

The exposure dose to the different VOCs was expressed in terms of the concentration of the most specific metabolite multiplied by the exposure duration (in terms of years).

The airborne concentration of the solvents in the mixture was monitored by means of Radiello samplers. The concentration of each VOC was correlated to its most specific urinary metabolite. As the workers wore full facepiece respirators during the entire work shift, biological monitoring was considered the most accurate method for assessing VOCs exposure. The urine samples were collected once per subject both before and after a work shift in June 2018.

The extraction and reading of the microRNA matrix were performed by Qiagen Genomic Services. The next-generation sequencing (NGS) analysis of the microRNAs was performed on samples of human blood plasma (200 μl). The collected readings were subjected to quality control, unique molecular index-based correction (to remove PCR replicates), alignment, and downstream analysis.

The microRNA data, normalized according to the Trimmed Mean of M values (TMM) method, were provided by QIAGEN. Genomic DNA was isolated from the whole blood of workers using the QiAmp DNA blood mini kit cat. N. 51306 (Qiagen, Germany). The hOGG1 Ser^326^Cys and the XRCC1 Arg^399^Gln gene polymorphisms were analyzed by Polymerase Chain Reaction (PCR) followed by restriction enzyme digestion (Fnu4HI and MspI, NEB), according to the methods described in a previous paper (19).

The hearing functionality was assessed by means of pure tone audiometry and high-resolution DPOAEs. All audiological measurements were performed in a quiet environment within the factory building, during a work break. The audiometric hearing level (HL), expressed in dB HL, was evaluated with steps of 5 dB at 11 frequencies in the 125–8,000 Hz range, using an Interacoustics AC40 audiometer, and the average level of the two ears was attributed to each subject. DPOAEs were measured using a dedicated acquisition system based on 24-bit NI-4461 PXI data acquisition boards, programmed in Labview 2015 (National Instruments, USA), driving two ER-2 loudspeakers and recording the response of an ER10B+ low-noise microphone (Etymotic Research, USA). High-resolution 2f_1_-f_2_ DPOAE complex spectra were recorded using chirp stimuli, with a constant primary frequency ratio *r* = f_2_/f_1_ = 1.22, stimulus levels (L_1_ and L_2_) = (61, 55) dB FPL, in the range f_DP_ = 1–4 kHz, with 20 Hz frequency resolution. The speed of the chirp stimuli was optimized to match the target frequency resolution of the analysis (20 Hz) to the temporal rate of change of the DPOAE frequency during the chirp. This way one can get a high-frequency resolution DPOAE spectrum in a relatively short time (3.75 s in this case). *N*=20 elementary DPOAE complex spectra were coherently averaged to increase the signal-to-noise ratio (SNR). Stimulus calibration in the ear canal of the forward pressure level (FPL) and evaluation of the emitted pressure level (EPL) of the DPOAE response was performed (20) to ensure test accuracy independent of the insertion depth of the probe in the ear canal. A time–frequency wavelet filtering method (21) was applied to unmix the DPOAE components, nonlinear distortion, and coherent reflection (22, 23), and to select the distortion or zero-latency (ZL) component. Canceling the contribution from coherent reflection has the 2-fold advantage of removing uncertainty associated with the amplitude fluctuations (up to 20 dB) known as the DPOAE fine structure, and significantly increasing the SNR. After unmixing, the ZL DPOAE level was computed in five third-octave frequency bands, and the average level of the two ears was attributed to each subject. In this study, for consistency with previous ones, we use f_DP_ to label the different frequency bands, reminding us that the corresponding f_2_ is given in this study by f_2_ = f_DP_/(2/r−1) = 1.564f_DP_.

Statistical analysis was performed using the SPSS/PC Statistical Software Package 25.0. (Inc., Chicago, IL, USA) and the Statistical Software R (R Foundation for Statistical Computing, Vienna, Austria).

The data normalized according to the TMM provided by QIAGEN were analyzed using Bioconductor routines. The differentially expressed genes in exposed and control groups were selected by means of the routine DESeq2 of the Bioconductor. The method used by the DESeq2 routine is based on the assumption that the reads counting for the *i*-th gene in the *j*-th sample (with j representing the subject index) is described by a GLM (generalized linear model) of the family of the Negative binomial distribution. The link function is a logarithmic one, relating the model coefficients to the log2 of the fold change between exposed and controls. A set of 56 genes with adjusted *p*-value ≤ 0.01 was selected in Sisto et al. (17).

A subset of microRNAs presumably associated with the auditory function was further selected in this study, as follows. The correlations between the 56 microRNA and the DPOAEs in six frequency bands were calculated. Only correlations with an absolute value higher than 0.3 were considered. A microRNA was considered associated with the auditory functionality if correlated with the DPOAEs at least in three different frequency bands. The same procedure was applied in the case of the audiometric HL at 11 standard octave and inter-octave frequencies between 125 and 8,000 Hz. This procedure yielded a subset of 12 microRNAs.

The mixed-effect linear regression models (lme) were studied to evaluate the association between the audiological variables and the microRNAs. Six and 11 models were studied, respectively, for the DPOAEs and the HL, in the different frequency bands. The mixed-effects models were chosen because the measurements in different frequency bands are obviously repeated measures on the same subject, treated as a random variable.

The subjects were also separated into two groups (defined normally hearing and hearing-impaired, with no reference to the standard audiometric definition of hearing impairment) according to their average HL (with a dichotomous threshold level arbitrarily set at 29 dB HL) and, independently, according to their average DPOAE level (with a dichotomous threshold level arbitrarily set at 16 dB EPL). Two different dichotomous criteria may be reasonably applied, because the DPOAE and HL levels, although strongly (negatively) correlated to each other, are differently sensitive to, respectively, peripheral and overall hearing damage.

The sparse partial least squares discriminant analysis (sPLS-DA) technique was applied to determine the combination of microRNA predictive variables maximizing the discrimination between the defined groups. If the maximum number of variables is set to *n*, starting from an initial space of dimension *N*, the components will be suitable linear combinations of *n* variables. For the sPLS-DA, an R package of the mixOmics library was used. Plotting both the cases and the microRNA in the 2-D space of the two main PLS components, it is possible to visually identify the microRNAs with a large component in the “direction” that best discriminates the two groups. The same sPLS-DA technique was applied to evaluate if the polymorphism of the investigated genes, hOGG1 and XRCC1, affects the susceptibility to hearing damage due to VOC exposure. For this purpose, the subjects were divided into two groups according to the hOGG1 and XRCC1 polymorphism. In the case of the hOGG1 polymorphism, the first group contains the *wt* variant, the second group contains both the *mut* and the *het* variants of the gene under investigation. In the case of the XRCC1, the first group contains both the *wt* and the *mut* variants, while the second group only contains the *het* variant. The reason for these different grouping criteria will be clarified later.

The auto contractive map (AutoCM for short), an unsupervised artificial neural network, developed by Semeion (24), was applied to detect hidden and nonlinear links among the data set variables. AutoCMs “spatialize” the correlation among variables by constructing a suitable embedding space where a visually transparent notion such as “closeness” among variables reflects accurately their associations. Its architecture is based on three layers of units: an input layer that captures the signal from the environment, a hidden layer that modulates the signal within the network, and an output layer that returns a response to the environment on the basis of the processing that occurred. Only one connection is established between the input layer and the hidden one, while this last is completely connected to the output layer. The variables of the dataset are scaled in the [0 1] interval and the variable and its complement to one are, indicated, respectively, with the prefixes “Max_” and “Min_”. The result of the AutoCM algorithm provides a 2d tensor matrix with the values of the connections between the hidden and the output layer at the end of the training. Each value of the matrix represents the strength of the connection between two variables, considering all the other variables together (third order of relationship). This matrix can be filtered and projected into a weighted graph by means of the Minimum Spanning Tree (MST), a graph that connects all the vertices (=variables), so that the lines (=relationships) are only those that are needed to interconnect all vertices with the shortest path (25). Spin Net is a new algorithm (25), based on a deep artificial neural network, created to exploit AutoCM's ability to understand the deep structure of the world of which the learned data is a part. When an incomplete input is introduced as input into the Spin Net system, the algorithm shows the path by which each variable tends to reach its stable value, interacting dynamically with all the other variables. Consequently, the number of the hidden layer of Spin Net depends on the number of cycles that Spin Net needs to reach equilibrium.

## Results

### Exposure

The personal A-weighted daily noise exposure level L_ex, 8h_, evaluated by means of personal phonometers, was 81.7 dB (A) on average for the roller painters and 87.2 dB (A) for the spray painters.

**Figure 1:**
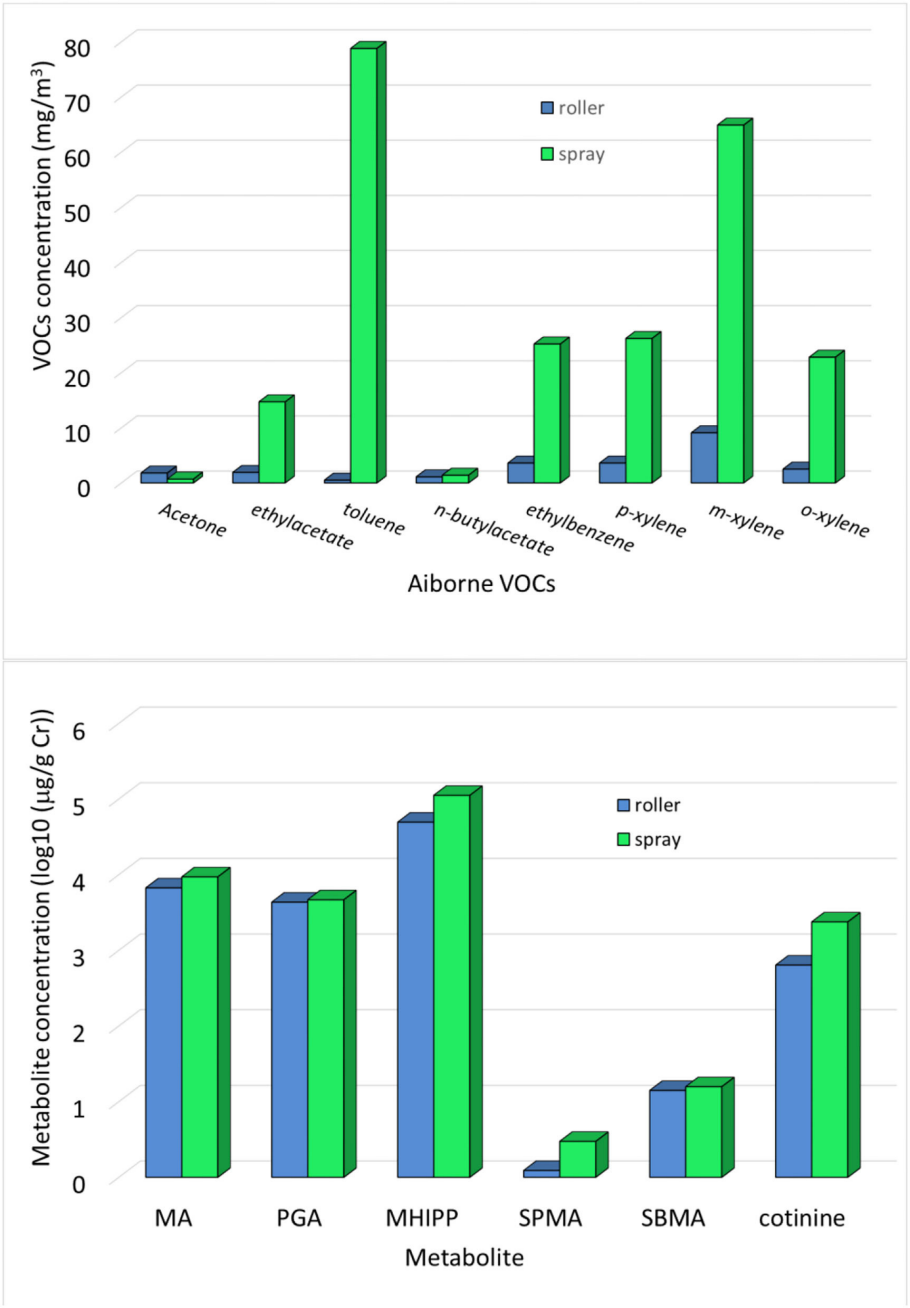
(Top) Airborne VOC concentrations and (bottom) metabolite concentrations measured in the end shift urine in the groups of roller and spray painters. To compare very different quantities, the metabolite concentrations were log-transformed.

The VOCs exposure in terms of airborne concentration (mg/m^3^) is shown in Figure 1 (top), where the data are separately shown for roller and spray painters. In the mixture, the highest concentrations are those of toluene and xylene. The VOCs exposure in terms of specific metabolites is also shown in Figure 1 (bottom). The total average concentration was: MA 7,523 μg/g Cr (max = 30,394, min = 1,072), PGA 4,441.9 μg/g Cr (max = 13,318.6, min = 1,246.3), MHIPP 68,974 μg/g Cr (max = 1,87,013, min = 13,996), SPMA 1.78 μg/g Cr (max 9.60, min = 0), SBMA 14.59 μg/g Cr (max = 30.15, min = 5.76), and cotinine 1,180.8 μg/g Cr (max = 9,773.5, min = 6.98).

The statistical associations between the VOCs metabolite concentrations and the DPOAE amplitudes were extensively discussed in Sisto et al. (3). In that study, significant associations were found between the DPOAEs and the VOCs metabolites with a negative slope, meaning that the higher the metabolite concentration, the worse the hearing function. With regard to the hearing thresholds measured by pure tone audiometry, they were found worse than they would have been expected as calculated with the ISO 1999 algorithms in terms of aging and noise level. Those findings supported the hypothesis that VOC exposure contributes to the exposure risk for hearing dysfunction, beyond age and noise exposure alone.

The 12 differentially expressed microRNAs that were also found correlated to the audiological variables are listed in Table 1. Note that, increasing HL means decreased hearing sensitivity, whereas increasing DPOAE level has the opposite meaning. Six microRNAs were found associated with the HL, and all correlations were negative. This means that the HL increase (i.e., damage) is associated with a downregulation of these microRNAs. Six different microRNAs were found associated with the DPOAE level, four negatively, and two positively. Table 1 provides a subset of differentially expressed microRNA that are plausible candidates for establishing significant regression laws with the audiological outcome variables. The results of the mixed model fits are shown in Table 2, where the β coefficients and their significance are reported. Note that, hearing impairment, as well as other diseases, may be expected to be associated with the upregulation of some microRNAs and downregulation of others. By comparing Tables 1 and 2, one may note that five microRNAs in Table 1 (out of 12) also yield a statistically significant correlation with DPOAE or HLs in specific frequency bands.

**Table 1:** List of the differentially expressed microRNAs significantly associated with the audiological variables, either HL or DPOAE level.

**microRNA name**	**Regulation (rel. to control group)**	**Log2-fold change**	***p*-value**	**Correlation sign**	**Audiological variable**
hsa-miR-885-3p	Downregulated	−3.72	1.09E-06	Negative	HL
hsa-miR-122-5p	Downregulated	−3.14	1.16E-05	Negative	HL
hsa-miR-195-5p	Downregulated	−1.82	3.19E-05	Negative	HL
hsa-miR-375	Downregulated	−2.72	8.34E-05	Negative	HL
hsa-miR-483-5p	Downregulated	−2.20	2.88E-03	Negative	HL
hsa-miR-193b-5p	Downregulated	−1.81	8.91E-03	Negative	HL
hsa-miR-497-5p	Downregulated	−2.64	7.56E-06	Negative	DPOAE
hsa-miR-206	Downregulated	−3.01	3.28E-05	Negative	DPOAE
hsa-miR-486-5p	Downregulated	−1.39	2.24E-03	Positive	DPOAE
hsa-miR-2355-5p	Upregulated	7.77	3.18E-03	Negative	DPOAE
hsa-miR-873-3p	Upregulated	7.74	6.09E-03	Negative	DPOAE
hsa-miR-92b-5p	Downregulated	−1.41	6.41E-03	Positive	DPOAE

The *p*-value refers to the comparison to a control group (17).

**Table 2:** Lme fit results for the audiological variables, audiometric hearing level (AHL), and DPOAE level (DP). The beta β coefficients are reported along with their standard error.

**Frequency (Hz)**	**microRNA name**	**β coeff (dB HL/counts)**	**β coeff (dB HL/counts) with** **hOGG1 polymorphism**
AHL 125	hsa-miR-195-5p	−2.407**(0.589)	
AHL 250	hsa-miR-195-5p hsa-miR-122-5p	−2.196**(0.506) −0.001*(0.0004)	
AHL 750	hsa-miR-122-5p	−0.001*(0.0005)	
AHL 1000	hsa-miR-195-5p hsa-miR-122-5p	−1.856***(0.447) −0.001**(0.0004)	
AHL 1500	hsa-miR-195-5p hsa-miR-122-5p	−1.955**(0.541) −0.002**(0.0005)	
AHL 2000	hsa-miR-195-5p hsa-miR-122-5p	−2.102**(0.676) −0.001* (0.001)	
AHL 3000	hsa-miR-195-5p	−2.212*(0.823)	
AHL 4000	hsa-miR-195-5p	−2.094*(0.832)	
DP 1153	hsa-miR-206 hsa-miR-92b-5p	−0.180*(0.080) 0.244*(0.114)	hOGG1 n.s.
DP 1452	hsa-miR-206 hsa-miR-92b-5p	−0.188*(0.068) 0.299*(0.098)	miR-206 −0.232* (0.064) hOGG1mut −7.57* (2.521) hOGG1wt −3.973 (1.93)
DP 1830	hsa-miR-497-5p	−1.866*(0.755)	miR-497-5p −2.166* (0.640) hOGG1mut −8.803*(3.022) hOGG1wt −1.700 (2.262)
DP 2305	hsa-miR-206 hsa-miR-92b-5p	−0.203**(0.067) 0.206*(0.095)	miR-206 −0.242* (0.060) hOGG1mut −6.578*(2.363) hOGG1wt −3.034 (1.830)

The statistical significance is coded:

^*^p < 0.05;

^**^p < 0.01;

^***^p < 0.001.

### sPLS-DA: Discrimination between groups sorted by DPOAE level and HL

**Figure 2:**
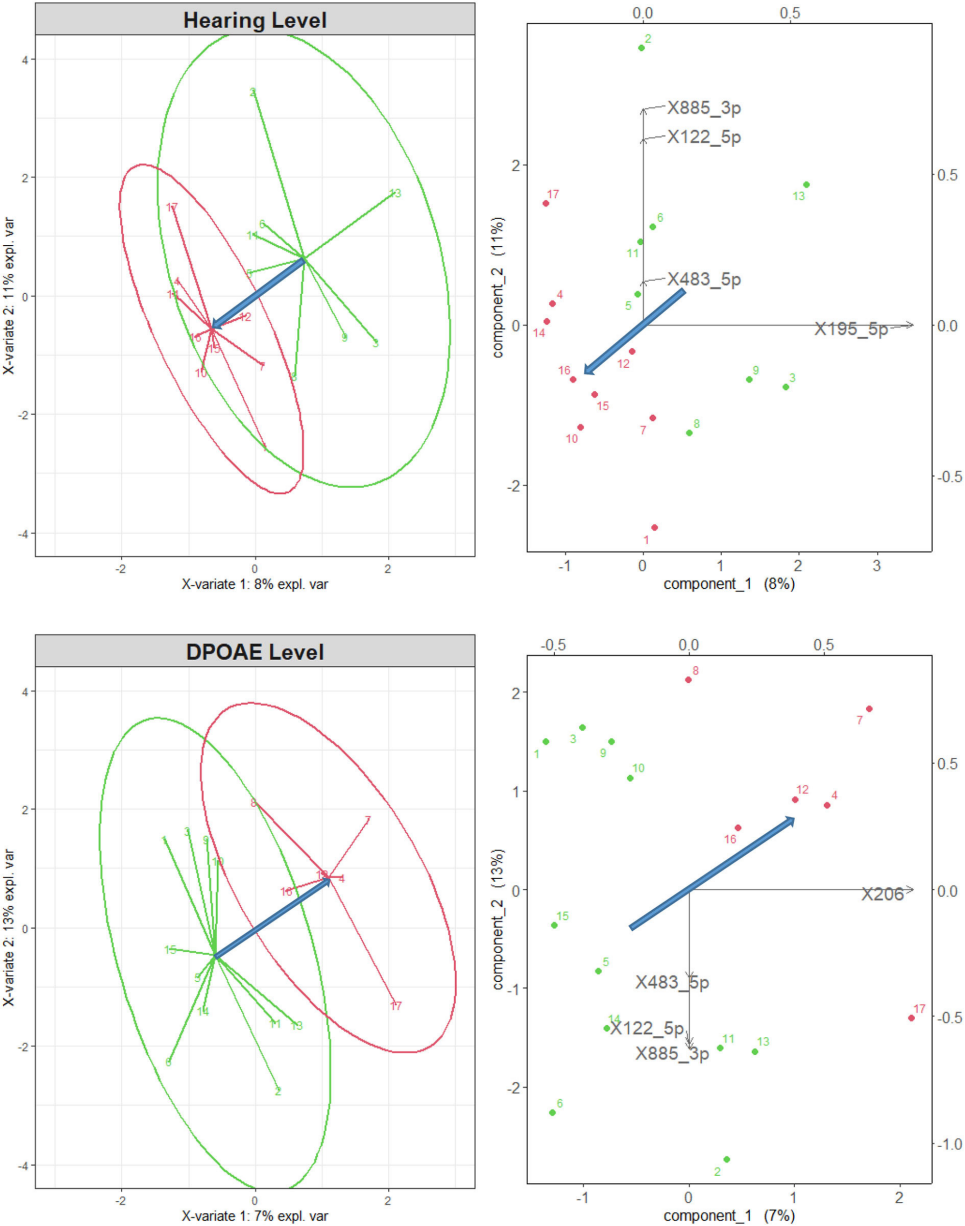
Left: sPLS-DA for normal-hearing subjects (green points and arrows) and impaired subjects (red), sorted according to their mean audiometric hearing (top) and DPOAE (bottom) level, considering all 56 differentially expressed microRNAs. The two groups are well separated. Right: biplot representation of the original variables in the 2d space of the transformed variables. Only the microRNAs with a correlation higher than 0.75 are shown by the black arrows. The blue arrows represent the direction of maximal discrimination between the two groups.

The groups sorted according to their average DPOAE level and HL were effectively discriminated by the sPLS-DA, using all *N* = 56 differentially expressed microRNAs, and *n* = 5 for each component, as shown in Figure 2. The green and red points represent, respectively, the coordinates of the normal-hearing and of the impaired subjects in the plane of the two main components (the linear combinations of the microRNA variables maximizing the discrimination between the two groups). The green and red arrows represent the differences with respect to the group average. The right and top axes are scaled by the variance of the data. In all panels, the blue arrows represent the direction of maximal discrimination between the two groups. In the biplots on the right-hand side of Figure 2, one can graphically evaluate the size and sign of the projection of the microRNA variables along the direction of maximal discrimination. In both cases, the microRNAs with the largest projection along the direction that best discriminates the two groups (and correlation >0.75) all belong to the subset of 12 microRNAs correlated with the audiological variables (see Table 1) and are among the most differentially expressed in the subjects exposed to VOCs, as found in Sisto et al. (17). Four of the selected microRNAs (miR195_5p, miR-885-3p, miR-122-5p, and miR483-5p) had been found to be correlated with the HL, while the miR-206 is the only one that had been found to be correlated with the DPOAE level, and, consistently with that negative correlation, has positive projection along the direction that points to the impaired group. In the HL case, all correlations were negative, and, indeed, the projections of miR195_5p, miR483-5p, miR-885-3p, and miR-122-5p all point to the direction of the normal group.

These results confirm the more quantitative results of the lme analysis shown in Table 2, in which the miR195_5p and miR-122-5p had been found negatively correlated with HL in selected frequency bands, and the miR-206 had been found negatively correlated with DPOAE level.

It is interesting to note that the miR483-5p, miR-885-3p, and miR-122-5p discriminate the audiological groups selected both by DPOAE level and by HL, whereas the miR-206 and the miR195_5p seem specifically related only to the discrimination based on, respectively, DPOAE level and HL. As a significant component of hearing loss induced by VOCs exposure may be of neural retrocochlear nature, therefore poorly associated with the DPOAE level, it would be interesting to further investigate this issue on a larger sample of subjects.

### sPLS-DA: Discrimination associated with hOGG1 and XRCC1 polymorphisms

**Figure 3:**
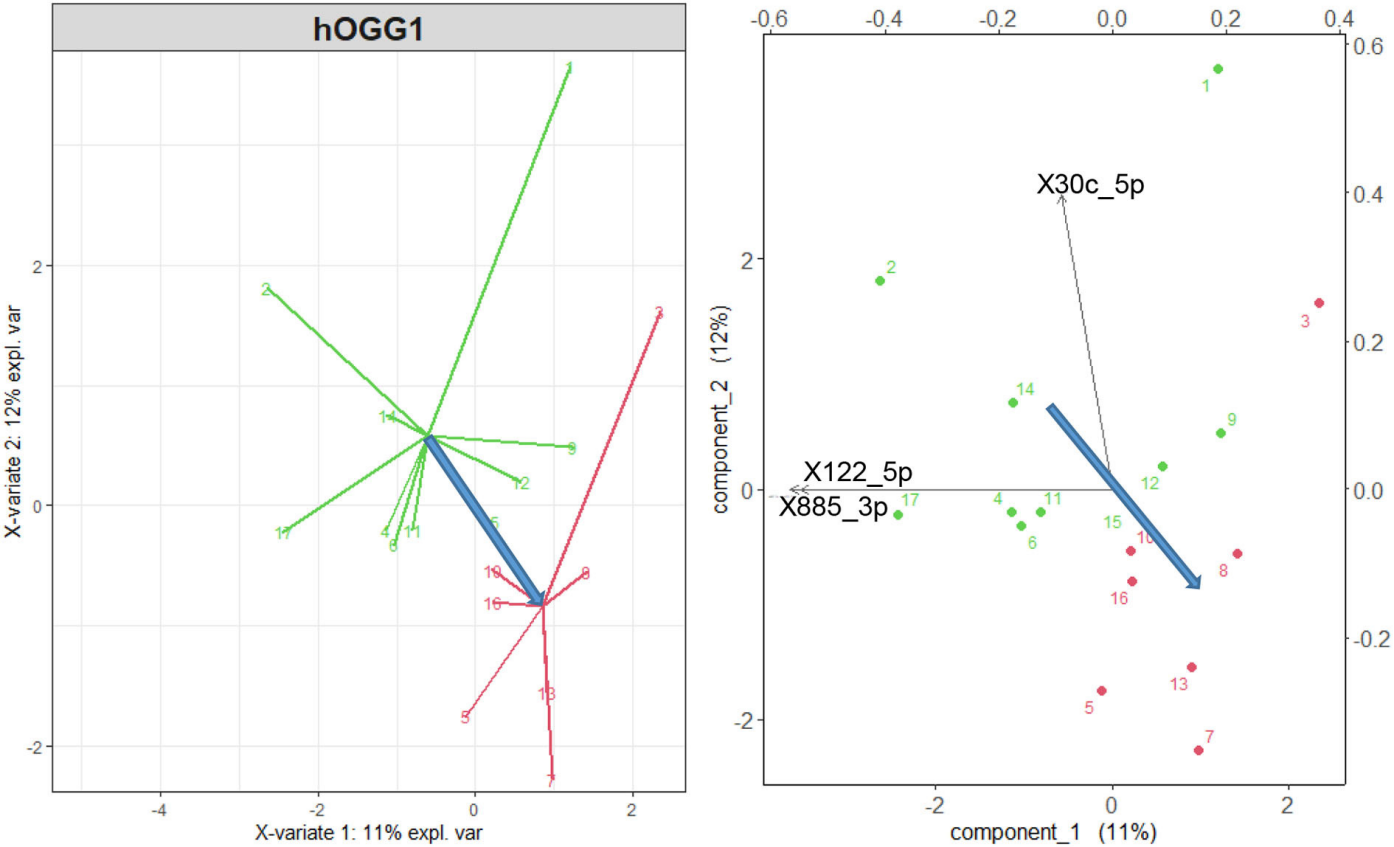
Left: sPLS-DA *wt* variant of the gene hOGG1 (green points and arrows) vs. *mut* and *het* variant (red), considering all the 56 differentially expressed microRNAs of Sisto et al. (17). Right: representation of the original variables in the 2d space of the transformed variables. Only the three microRNAs with a correlation higher than 0.75 are shown by the black arrows. The blue arrows represent the direction of maximal discrimination between the two groups.

As shown in Figure 3 (left), the *wt* group (green points and arrows) and that including the *mut* and *het* variants of the hOGG1 polymorphism (red) are discriminated by the microRNA variables. Although this discrimination is not satisfactory from the point of view of sensitivity and specificity, the biplot of Figure 3 (right), in which both cases and original variables are represented in the space of the transformed variables shows the direction of maximum discrimination between the two groups of hOGG1 polymorphism, indicated by the blue arrow.

Note that the miR-885-3p and miR-122-5p, which point in the direction of the *wt* group, are the most significantly differentially expressed microRNAs correlated with HL in Table 1 and that the miR-122-5p is also significantly correlated with HL in selected frequency bands (see Table 2). This suggests a disadvantageous role of the hOGG1 *mut* and *het* variants, as regards the risk of hearing impairment due to the exposure to VOCs, as also confirmed by the multivariate linear regressions using the hOGG1 polymorphism as a two-level factor, as shown in the last column of Table 2 for miR-497_5p and miR-206, both correlated, in this case, with the DPOAE level.

**Figure 4:**
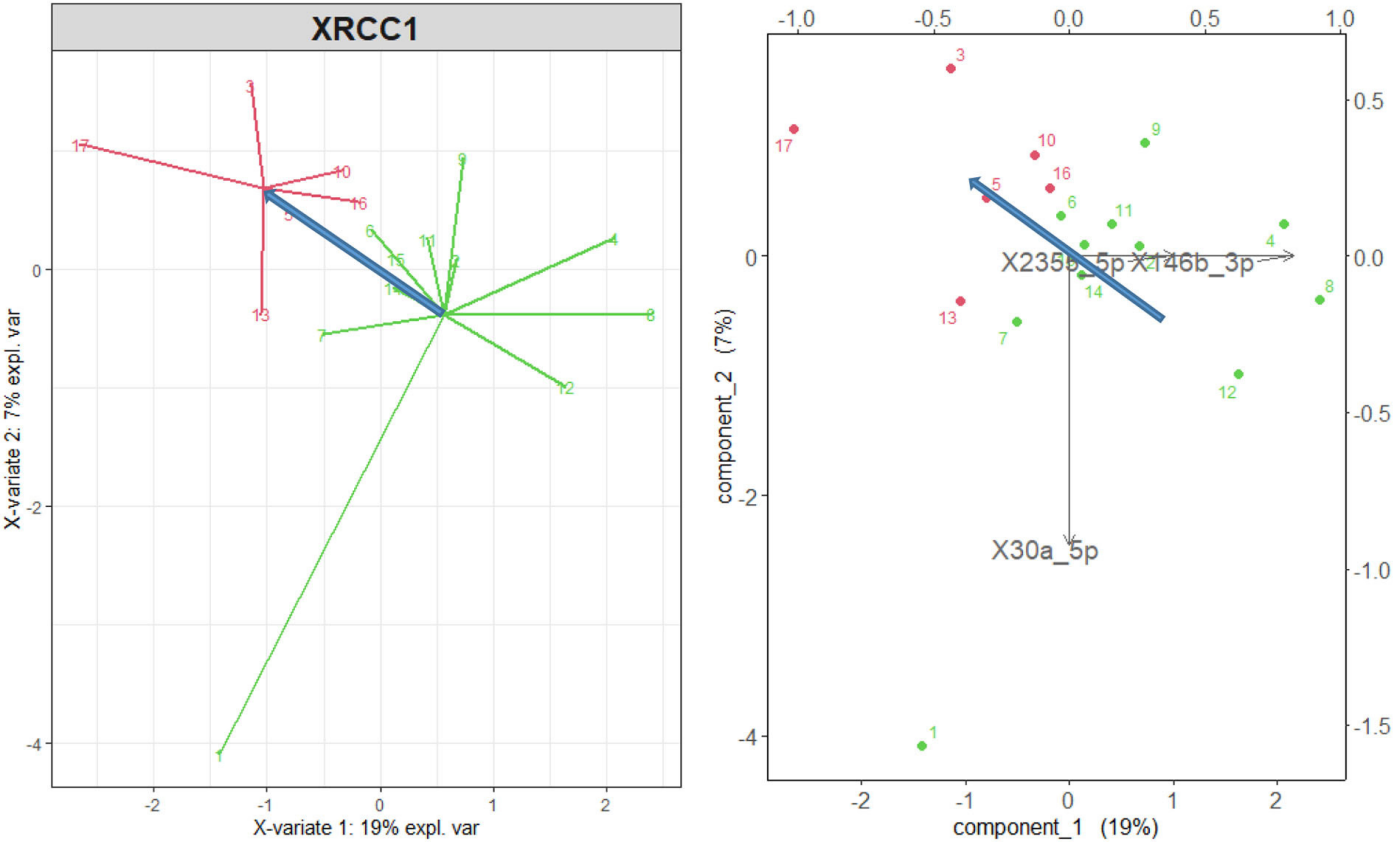
sPLS-DA *wt* and *mut* variants of the gene XRCC1 (green points and arrows) vs. *het* variant (red). Right: representation of the original variables in the 2d space of the transformed variables. The microRNA with a correlation higher than 0.75 are shown. The blue arrows represent the direction of maximal discrimination between the two groups.

The microRNA variables also discriminate effectively against the XRCC1 variants, and Table 1 shows that the miR2355_5p, which points in the direction opposite to the *het* variant in Figure 4, is correlated with the DPOAE level. Nevertheless, in this case, the correlation is not sufficient to yield significant regressions with any DPOAE band level in Table 2.

### Machine learning algorithm

**Figure 5:**
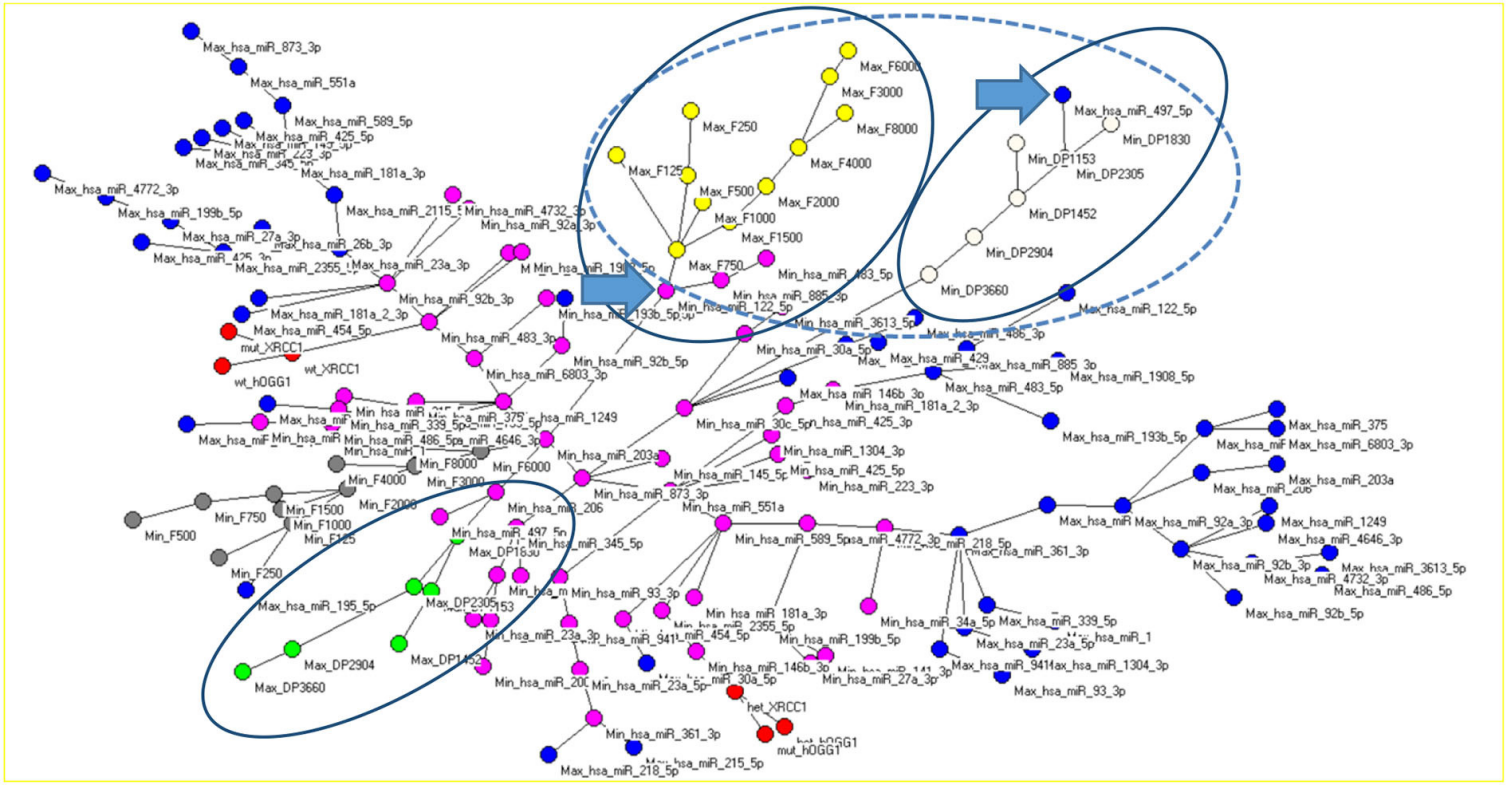
MST Graph, visually representing the most relevant connections between all the considered variables, microRNA (blue and pink circles, respectively, for the normalized variable and its complement to unity), HL (yellow and gray circles), DPOAE (green and white circles), and polymorphisms (red circles). The ellipses encircle branches of audiological variables correlated to each other. The blue arrows point to microRNAs that are strongly associated with specific branches of the audiological variables. The dashed-line ellipse highlights the negative correlation between HL and DPOAE levels.

In Figure 5, the MST Graph (which selects and displays the most significant relationships identified by the AutoCM Neural Network) is reported. The HL variables are labeled by F followed by the frequency, and they are strongly related to each other. The same applies to the DPOAE levels, labeled by DP followed by the band center frequency.

The negative correlation between the HL and miR-122-5p and that between the DPOAE level and miR-497-5p are well represented in Figure 5. As indicated by the arrows, the two microRNAs are directly connected to the two trees of HL and DPOAE level. The miR-122-5p is evidently negatively correlated to the HL; in the graph, in fact, Min_miR-122-5p is located in a branch directly connected with the Max_ audiometric variables. When the Spin Net query is launched, the activated variables are listed in Table 3 (A1). One can see how miR-122-5p in its Min_ value activates, among the highest values, the variables F in their Max_ value, e.g., Max_F1500 and Max_F750. With regard to the miR-195-5p, although this variable is not close to the HL in the MST Graph, the Spin Net algorithm was able to individuate a mutual activation of these variables. The activation degree of the variables connected to the miR-195-5p is shown in Table 3 (A5). In this table, one can see how miR-195-5p, in its Min_ value, activates among the highest values of the variables F in their Max_ value, e.g., Max_F125 and Max_F250.

**Table 3:** The variables on which the query with Spin Net was carried out are marked with two asterisks (**): Min_has_miR_122-5p (A1), Min_hsa_miR_92b_5p (A2), Max_hsa_miR_497_5p (A3), Min_hsa_miR_206 (A4), and Min_hsa_miR_195_5p (A5).

**A1**		**A2**		**A3**		**A4**		**A5**	
**Variables**	**Act**.	**Variables**	**Act**.	**Variables**	**Act**.	**Variables**	**Act**.	**Variables**	**Act**.
Min_hsa_miR_122_5p**:	0.938	Min_hsa_miR_486_5p:	0.943	**Min_DP2305:**	0.956	Min_hsa_miR_375:	0.940	Min_hsa_miR_195_5p**:	0.948
Min_hsa_miR_885_3p:	0.932	Min_hsa_miR_92b_5p**:	0.942	Max_hsa_miR_497_5p**:	0.954	Min_hsa_miR_206**:	0.938	**Max_F125:**	0.946
Min_hsa_miR_429:	0.930	Min_hsa_miR_4732_3p:	0.941	Max_hsa_miR_34a_5p:	0.953	Min_hsa_miR_497_5p:	0.938	**Max_F250:**	0.943
Min_hsa_miR_193b_5p:	0.929	Min_hsa_miR_92b_3p:	0.939	**Min_DP1452:**	0.946	Min_F6000:	0.936	Min_hsa_miR_215_5p:	0.942
Min_hsa_miR_497_5p:	0.929	Min_hsa_miR_92a_3p:	0.937	**Min_DP1830:**	0.946	Min_hsa_miR_6803_3p:	0.933	Min_hsa_miR_141_3p:	0.941
Min_hsa_miR_483_5p:	0.927	Min_hsa_miR_125b_5p:	0.937	Max_hsa_miR_429:	0.937	Min_F8000:	0.932	**Max_F1000:**	0.938
Min_hsa_miR_30a_5p:	0.926	Min_hsa_miR_339_5p:	0.934	Max_hsa_miR_885_3p:	0.924	Min_hsa_miR_483_3p:	0.931	Max_hsa_miR_200a_3p:	0.937
Min_hsa_miR_34a_5p:	0.925	Min_hsa_miR_486_3p:	0.933	Max_hsa_miR_483_5p:	0.923	**Max_DP1830:**	0.931	**Max_F1500:**	0.937
Min_hsa_miR_1908_5p:	0.924	Min_hsa_miR_193b_5p:	0.933	Max_hsa_miR_206:	0.907			Min_hsa_miR_92b_5p:	0.933
**Max_F1500**:	0.923	Min_hsa_miR_4646_3p:	0.932	Max_F4000:	0.905			Min_hsa_miR_375:	0.932
Min_hsa_miR_218_5p:	0.923	Min_hsa_miR_195_5p:	0.932						
**Max_F750:**	0.923	Min_hsa_miR_6803_3p:	0.931						
**Max_F500:**	0.923	Min_hsa_miR_483_3p:	0.930						
Min_hsa_miR_375:	0.922	**Min_DP1452:**	0.930						
Min_hsa_miR_1:	0.922	Min_hsa_miR_1249:	0.929						
**Max_F250:**	0.921	Min_hsa_miR_1908_5p:	0.929						
**Max_F1000:**	0.921	Min_hsa_miR_1:	0.927						
**Min_F6000:**	0.921	**Min_DP2904:**	0.926						

The variables of main interest for this study are printed in bold.

The anti-correlation between the miR-497-5p and the DPOAEs is also evident in Figure 5. The Spin Net query showed the connections listed in Table 3 (A3), where we can see how miR-497-5p, in its Max value, activates among the highest values the DP variables in their Min value, e.g., Min_DP2305 and Min_DP1452. Finally, in Table 3, (A4) and (A2), respectively, reported the activation of the variables in the case of miR-206 and miR-92b-5p. The first is negatively correlated to the DPOAEs (e.g., Max_DP1830), while the second is positively correlated to the DPOAEs (e.g., Min_DP1452).

The MST Graph also motivated the choice of differently grouping the variants in the hOGG1 and XRCC1 cases. In the hOGG1 case, the *mut* and *het* variants of the hOGG1 belong to the same tree of connections, so they were grouped together and compared to the *wt*. In the case of the XRCC1, the *wt* and *mut* belong to the same tree of connections. This graphical evidence was interpreted as suggesting that in this case the *wt* and *mut* variants are connected to each other, so they were grouped together and compared to the *het* variant.

## Discussion

In this study, statistically significant associations were found between audiological variables, i.e., HLs and DPOAE levels, and specific microRNAs that, in a previous study (17), had been found differentially expressed in workers exposed to VOCs and in a control group. The microRNAs significantly associated with the HLs and those associated with the DPOAEs are different, suggesting dissimilar damage effects on the peripheral and central auditory systems. Two microRNAs were found significantly associated with the hearing thresholds, miR-122-5p, which is one of the dominant microRNAs found in hepatocytes (26), and microRNA-195-5p which might be implicated in several neurodegenerative disorders (27, 28).

In both cases, an increase in hearing impairment, i.e., a higher HL, is associated with a downregulation of the considered microRNAs. This circumstance seems to be coherent with the fact that the miR-122-5p and miR-195-5p were found downregulated in the exposed workers with respect to the controls. The statistical association between microRNA and HL is particularly strong in the low-mid frequency range, with no significant association between the aforementioned microRNAs and HL found at frequencies higher than 4,000 Hz. This is consistent with the observation that the hearing damage caused by solvents especially occurs in the low-medium frequency range. The microRNAs associated with DPOAEs are miR-206, miR-497-5p, and miR-92b-5p. In the case of miR-206, the decrease of the DPOAE amplitude, suggesting a reduction of the cochlear amplifier effectiveness, is associated with an upregulation of the microRNA. Similarly, miR-497-5p is significantly associated with the DPOAE in the band 1,830 Hz, while the DPOAE level is positively associated with miR-92b-5p. Several papers have shown the effect of miR-206 on the nervous system and neurological diseases, such as Amyotrophic Lateral Sclerosis (ALS) and Spinal Muscular Atrophy (SMA) [e.g., (29)]. This microRNA was abnormally expressed in animal models of ALS and over-expressed in patients when compared to controls, while in a mouse model, miR-206 has been found to delay ALS, thus promoting the regeneration of neuromuscular synapses (30). In a recent study, the authors assessed that early modulation of microRNA-206 expression could delay SMA neurodegenerative pathway, proposing microRNA-206 as an interesting and novel target for SMA therapy (31). miR-92b is over-expressed specifically in brain primary tumors and neuronal-specific stem cells, showing dynamic expression patterns in the developing brain (32). Studies on miR-497-5p show that it is downregulated in several tumor types, acting as a tumor suppressor, but the functional role of this microRNA is still bivalent, behaving as both a promoter and a suppressor (33).

Furthermore, different studies have demonstrated the relationship between genetic factors, including DNA synthesis-related genes, DNA repair pathways such as base excision repair, and individual susceptibility of workers exposed to industrial noise (34). Mutations that occur in base excision repair genes (i.e., hOGG1 and XRCC1) may affect the normal repair functions resulting in a lower DNA repair capacity and an increase in the probability of developing noise-induced hearing loss (34). In this respect, Shen and coworkers (35) investigated the genotyping of 615 workers with noise-induced hearing loss and 615 normal-hearing workers in China showing that the hOGG1 Ser326Cys polymorphism may modify the susceptibility to noise-induced hearing loss (35). These data underline that exon 7 of Ser326Cys hOGG1 gene polymorphism may modify the activity of the enzyme, resulting in lower DNA damage repair efficiency, while the hOGG1 Cys/Cys *mut* mutant genotype has been found statistically associated with noise-induced hearing loss. In the study mentioned above, a higher number of subjects have been found with the hOGG1 Cys/Cys genotype among noise-induced hearing loss workers compared to normal hearing workers, suggesting that the hOGG1 Cys/Cys genotype might be particularly susceptible to the risk of noise-induced hearing loss (35). It was also highlighted that the hOGG1 Ser326Cys polymorphism had a synergistic effect when combined with other factors such as noise, exposure level, time, and smoking habit, as found in our present study regarding the co-exposure to noise and ototoxic solvents (35).

The results of this study suggest that the *mut* and *het* hOGG1 genotypes may indeed contribute to the susceptibility to hearing damage of workers exposed to noise and ototoxicants. Such susceptible biomarkers are useful in the biomonitoring of the hearing impairment in workers simultaneously exposed to noise and ototoxic solvents. Further investigations and data analysis are needed, involving a larger number of workers compared to healthy controls.

## Conclusion

The statistical association of audiological variables with microRNA expression was studied, using different statistical analysis techniques. Both HLs, measured by pure tone audiometry, and DPOAE, an objective biomarker of the cochlear amplifier effectiveness, are significantly associated with microRNA expression levels. The HL shifts were found mainly associated with the downregulation of miR-122-5p and miR-195-5p. The DPOAE levels were found mainly associated with the miR-206, miR-497-5p, and miR-92b-5p. Only in the case of the miR-206, the reduced functionality of the cochlear amplifier was found associated with an upregulation of the microRNA, while in the case of the miR-497-5p and miR-92b-5p, the cochlear dysfunction is related to the downregulation of the microRNAs. The association between the microRNAs and the audiological variables was found significant in the low-mid frequency range. A significant association was found between the microRNAs and the polymorphisms of the hOGG1, responsible for coding enzymes involved in DNA repair. A set of microRNAs was found under-expressed in the *mut* and *het* groups, with respect to the *wt*. These microRNAs are negatively correlated to the HL, suggesting that the *mut* and *het* variants are disadvantaged with respect to the *wt*.

This paper could give a contribution to the identification of microRNAs as specific biomarkers of susceptibility in the case of exposure to ototoxic and, more generally, neurotoxic substances. Monitoring these biomarkers could help understand mechanistically the toxicity effects on exposed workers, and design prevention strategies focused on these susceptibility indexes.

## Data availability statement

The raw data supporting the conclusions of this article will be made available by the authors, without undue reservation.

## Ethics statement

The studies involving human participants were reviewed and approved by ASUR Marche Prevention Department. The patients/participants provided their written informed consent to participate in this study.

## Author contributions

RS: conceptualization and data analysis and miRNA data analysis. GT, FS, LC, and RS: data collecting. RS, AM, GM, and SB: statistical data analysis. PB and GM: machine learning algorithms. PC and PC: polymorphisms analysis. RS, PC, and AM: manuscript writing. All the authors reviewed and approved the manuscript.

## Funding

This work was supported by Grant BRIC ID09 PAR 2019–2021 from the INAIL Research.

## Conflict of interest

The authors declare that the research was conducted in the absence of any commercial or financial relationships that could be construed as a potential conflict of interest.

## Publisher's note

All claims expressed in this article are solely those of the authors and do not necessarily represent those of their affiliated organizations, or those of the publisher, the editors and the reviewers. Any product that may be evaluated in this article, or claim that may be made by its manufacturer, is not guaranteed or endorsed by the publisher.
